# Hydrogen treatment-improved uniform deposition of Ag nanoparticles on ZnO nanorod arrays and their visible-light photocatalytic and surface-enhanced Raman scattering properties

**DOI:** 10.1186/1556-276X-8-325

**Published:** 2013-07-16

**Authors:** Sio-Le Lin, Kai-Chih Hsu, Chih-Hsiung Hsu, Dong-Hwang Chen

**Affiliations:** 1 Department of Chemical Engineering, National Cheng Kung University, Tainan 701, Taiwan, Republic of China

**Keywords:** ZnO nanorod arrays, Hydrogen treatment, Ag nanoparticles, Photocatalytic, Surface-enhanced raman scattering

## Abstract

ZnO nanorod arrays were synthesized by chemical bath deposition. After heat treatment in hydrogen or air, Ag nanoparticles were deposited on ZnO nanorod arrays by photo-reduction method. The size of Ag nanoparticles as well as the surface morphology, structure, composition, and optical property of ZnO nanorod arrays before and after the deposition of Ag nanoparticles were characterized by SEM, XRD, EDS, and UV/VIS/NIR spectrophotometer. As compared to the samples with heat treatment in air or without heat treatment, the ZnO nanorod arrays after heat treatment in hydrogen allowed Ag nanoparticles to be deposited more uniformly, densely, and numerously. Also, they exhibited higher efficiency for the visible light-driven photocatalytic degradation of Rhodamine 6G (R6G) dye. The effects of the amount of Ag nanoparticles, initial dye concentration, and temperature on the photocatalytic degradation efficiency were investigated. Furthermore, they also exhibited better surface-enhanced Raman scattering property for the detection of R6G dyes.

## Background

Nowadays, environmental problems relating to wastewaters are becoming much more serious than ever, and the photocatalytic technique with metal oxide semiconductors has become one of the most promising methods for wastewater treatment [1-6]. Among various metal oxide semiconductors, ZnO has gained pretty much attention with respect to the degradation of various pollutants owning to its high photosensitivity, high catalytic efficiency, low cost, non-toxicity, environmental sustainment stability, and wide band gap [7,8]. However, due to its wide band gap, ZnO can only be activated by ultraviolet light of wavelength below 385 nm, only accounting for less than 5% of the solar energy, which practically limits the use of solar light or visible light. Furthermore, energy saving consideration is now being more regarded. How to extend the photo response of ZnO toward the visible spectral region is now being an important issue [7]. To solve this tough problem, ZnO modification has been extensively explored, such as combining with other semiconductors, doping and coating with noble metals, and modifying with organic polymers [9-17]. Many researchers have reported the synthesis of Ag/ZnO composites and their applications in various fields, especially in photocatalytic degradation of organic dyes [18-34] and surface-enhanced Raman scattering (SERS) [18,35-37]. Silver metal exhibits plasmon resonances under visible light; moreover, it is stable, non-toxic, easy to synthesize, and relatively cheap compared to other noble metals. Therefore, combining silver metals with ZnO can effectively help the use of visible light.

In this work, we presented a method to synthesize silver-coated ZnO nanorod arrays with silver nanoparticles depositing uniformly onto top, side, and bottom of nanorods, which offered much more active sites to take part in photocatalysis. The effect of heat treatment in hydrogen or air on the deposition of Ag nanoparticles on ZnO nanorod arrays was examined. After the photocatalysts were successfully obtained, we used Rhodamine 6G (R6G) as the target containment and visible light as the light source to investigate the photocatalytic activity of silver-coated ZnO nanorod arrays. The effects of the amount of Ag nanoparticles, initial R6G concentration, and temperature on the photocatalytic degradation efficiency were investigated.

In addition to photocatalysis, Ag/ZnO can also be used in SERS, which is an extraordinary analytical tool for determining chemical and biological information of molecules on solid substrates and can provide unique fingerprints of analytes, making its rapid development since it appeared [38-47]. So, in this work, R6G was also used as the detection target for the study on the SERS property of silver-coated ZnO nanorod arrays. The effect of heat treatment in hydrogen or air on the influence of SERS performance was investigated. The detection limit of R6G was also determined.

## Methods

Sodium hydroxide and 2-methoxyethanol were obtained from Fluka (Fluka Chemical Corporation, St. Louis, Milwaukee, WI, USA). Zinc acetate and zinc nitrate were purchased from J.T. Baker Chemical Company (Phillipsburg, NJ, USA). Diethylenetriamine (DETA) was the product of Riedel-DeHaen (Honeywell International, Inc., Morristown, NJ, USA). Silver nitrate 99.9% was the product of Alfa Aesar (Ward Hill, MA, USA). Rhodamine 6G and monoethanolamine (MEA; 99.5%) was obtained from Sigma-Aldrich Corporation (St. Louis, MO, USA). The water used throughout this work was the reagent grade water produced by a Milli-Q SP ultra-pure-water purification system of Nihon Millipore Ltd., Tokyo, Japan.

ZnO nanorod arrays were prepared according to our previous work on the synthesis of Al-doped ZnO nanorod arrays but without Al doping [48,49]. Firstly, 0.5 ml MEA was added to a solution containing 11 ml 2-methoxyethanol and 1.8 g zinc acetate, which formed the ZnO sol–gel solution. ZnO seed layer was prepared by spin coating the sol–gel solution (0.1 ml) on a glass substrate (2.5 cm × 2.5 cm) at a rotation speed of 3,000 rpm for 30 s, and the films were then annealed at 350°C for 10 min. The step mentioned above was repeated eight times, and the acquired ZnO thin films were then annealed to 550°C for 2 h to get the final ZnO seed layer. Secondly, the ZnO seed layer was placed in an autoclave containing growth solution consisting of 30 ml water, 1.32 g zinc nitrate, 0.46 ml DETA, and 0.8 ml NaOH. After that, the growth solution was heated to 95°C for 6 h to get the ZnO nanorod arrays, which was noted as ZnO. The ZnO nanorod arrays were annealed in Ar/H_2_(97/3) or air atmosphere at 400°C for 2 h to get ZnO-H and ZnO-A, respectively.

For the deposition of Ag nanoparticles on ZnO, ZnO-H, and ZnO-A, the resultant ZnO, ZnO-H, and ZnO-A were immersed in an aqueous solution of AgNO_3_ (5 ml, 0.01 M) and were illuminated under UV light (λ = 254 nm) for 10 min. This step was repeated three times to get ZnO@Ag, ZnO-H@Ag, and ZnO-A@Ag. For the investigation on the effect of Ag content on the photocatalytic activity of ZnO-H@Ag, the deposition step was conducted for 4 min × 1, 7 min × 1, 10 min × 1, 10 min × 2, 10 min × 3, or 10 min × 4 (here × denotes the repeating time).

The surface morphology and energy dispersive X-ray (EDX) spectroscopy were observed by a high-resolution field emission scanning electron microscopy (HR-FESEM, JEOL SEM 6700F; JEOL Ltd., Tokyo, Japan). The crystalline structure was characterized by X-ray diffraction (XRD) analysis on a Rigaku D/max-ga X-ray diffractometer (Rigaku Corporation, Tokyo, Japan) at 40 kV with Cu K_α_ radiation (λ = 0.1542 nm).

For the photocatalytic degradation of R6G, the photocatalysts (1.25 cm × 1.25 cm, ZnO, ZnO-H, ZnO-A, ZnO@Ag, ZnO-H@Ag, or ZnO-A@Ag) were placed into 5 ml of R6G solution, allowed to equilibrate for 30 min in the darkness, and then followed by the lamp’s (200 W, λ > 400 nm, manufactured by Oriel Instruments Corporation, Stratford, CT, USA) switching up to initiate the reaction. During the reaction, a certain amount of solution was withdrawn at certain reaction intervals to determine the remaining concentration of R6G by a JASCO model V-570 ultraviolet–visible near-infrared (UV/VIS/NIR) spectrophotometer (JASCO Analytical Instruments, Easton, MD, USA) at 527 nm.

For the study on the SERS property, the substrates (ZnO, ZnO-H, ZnO@Ag, ZnO-H@Ag, and ZnO-A@Ag) were immersed in the 40 ml of R6G solutions with different concentrations for 40 min and were then analyzed by the micro-Raman spectrometer (Scinco, 532 nm; Scinco Co., Ltd. Kangnam-Gu, Seoul, South Korea) to get the SERS spectra of R6G.

## Results and discussion

**Figure 1:**
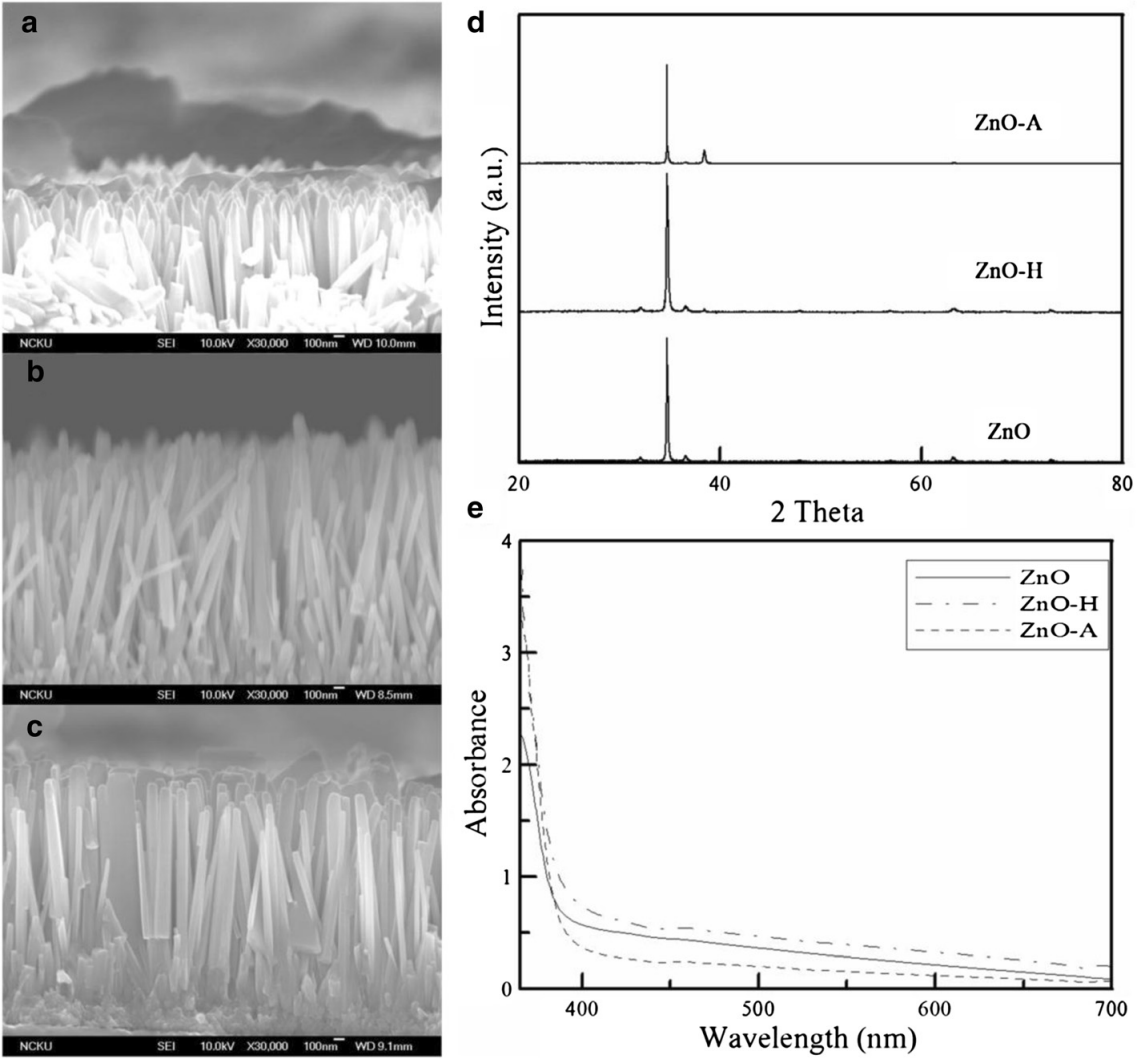
**SEM images, XRD patterns, and UV–vis absorption spectra of ZnO, ZnO-H, and ZnO-A.** SEM images of **(a)** ZnO, **(b)** ZnO-H, and **(c)** ZnO-A. XRD patterns **(d)** and UV–vis absorption spectra **(e)** of ZnO, ZnO-H, and ZnO-A.

Figure 1a,b,c shows the cross-sectional scanning electron microscopy (SEM) images of ZnO, ZnO-H, and ZnO-A. It was obvious that they have all grown perpendicular to the glass substrate and revealed that the heat treatment in Ar/H_2_(97/3) or air atmosphere did not significantly change or destroy the one-dimensional structure of ZnO nanorod arrays. From the EDX analysis, the atomic percentages of oxygen in the ZnO, ZnO-H, and ZnO-A were determined to be 52.9, 51.6, and 56.5, respectively. This revealed that the heat treatment in Ar/H_2_(97/3) slightly resulted in the increase of oxygen vacancy while that in air atmosphere led to the decrease of oxygen vacancy. Although the atomic percentages of oxygen in ZnO, ZnO-H, and ZnO-A acquired by EDX were semi-quantitative, they at least could reflect the variations of oxygen atomic percentages in ZnO, ZnO-A, and ZnO-H. The variations were reasonable based on the principle of heat treatment. As for the result that the atomic percentages of oxygen were larger than zinc in all the three ZnO nanorods, this phenomenon was similar to some works on the doped or undoped ZnO [50,51]. Furthermore, the XRD patterns of ZnO, ZnO-H, and ZnO-A as shown in Figure 1d indicated that they all exhibited the strong characteristic peak for the (002) plane of wurtzite-type ZnO (hexagonal) around the scattering angle of 35°, revealing the heat treatment in Ar/H_2_(97/3) or air atmosphere did not significantly change the crystalline structure of ZnO. In addition, the absorption spectra were shown in Figure 1e. They all had no significant absorption in the visible light region. Also, in the visible light region, the heat treatment in Ar/H_2_(97/3) or air atmosphere led to the only slight increase or decrease of absorption, respectively.

**Figure 2:**
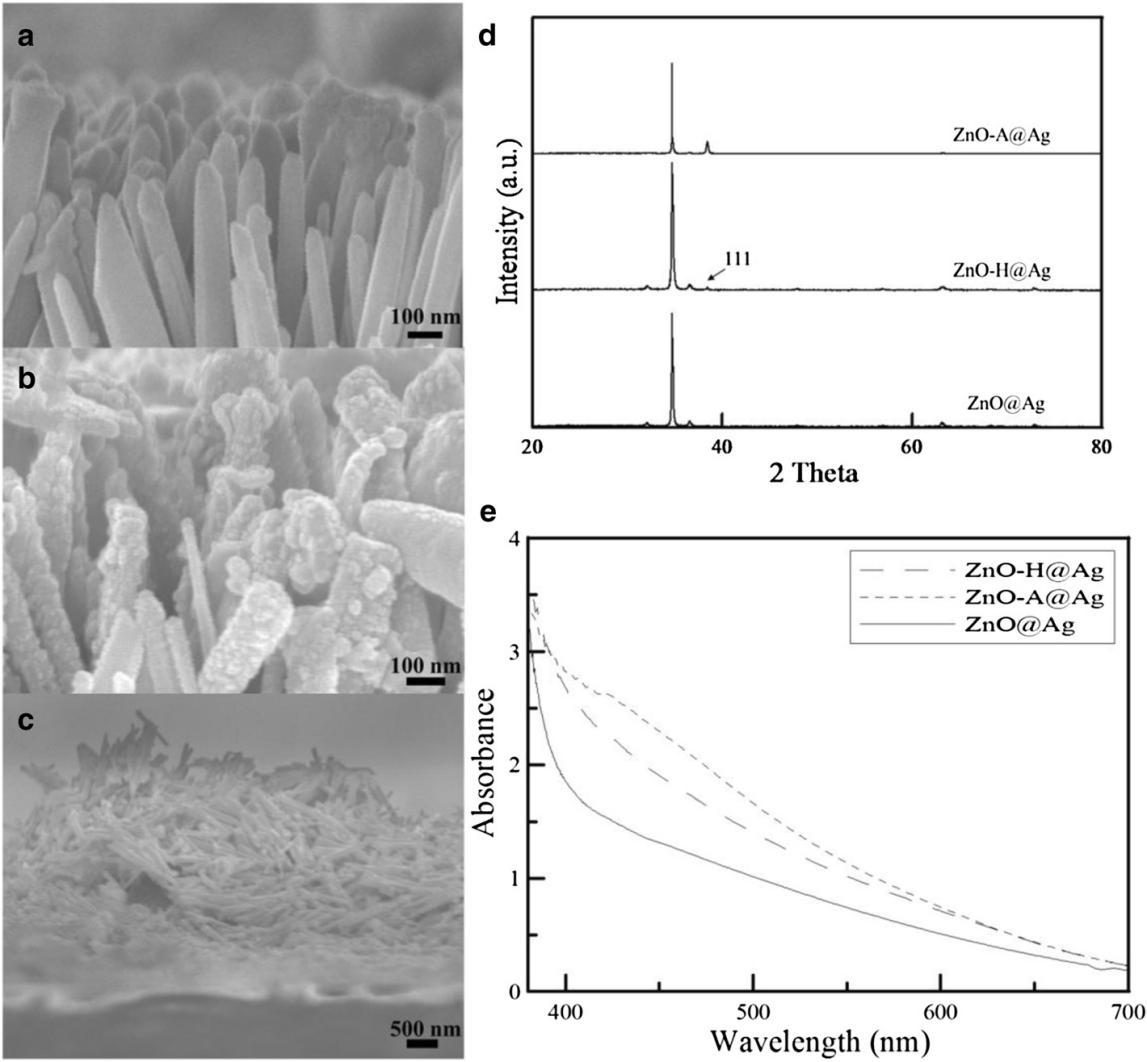
**SEM images, XRD patterns, and UV–vis absorption spectra of ZnO@Ag, ZnO-H@Ag, and ZnO-A@Ag.** SEM images of **(a)** ZnO@Ag, **(b)** ZnO-H@Ag, and **(c)** ZnO-A@Ag. XRD patterns **(d)** and UV–vis absorption spectra **(e)** of ZnO@Ag, ZnO-H@Ag, and ZnO-A@Ag.

Figure 2a,b,c shows the cross-sectional SEM images of ZnO@Ag, ZnO-H@Ag, and ZnO-A@Ag. For ZnO@Ag, Ag nanoparticles tended to deposit onto the top of nanorods. A similar phenomenon has been observed and could be explained as follows [36,52]: Because of the electronegativity difference between Zn and O, there were electric fields forming within ZnO nanorods whose top and bottom were related to the lowest unoccupied molecular orbital (LUMO) and highest occupied molecular orbital (HOMO), respectively. When ZnO nanorods were illuminated by UV light, the electrons tend to be excited from the bottom to the top and thus the top of nanorods always accumulated more electrons, which could reduce silver ions to form silver nanoparticles easily. For ZnO-H@Ag, Ag nanoparticles deposited uniformly on the top, side, and bottom of the ZnO nanorods with hydrogen treatment. This could be explained by two reasons: (1) after hydrogen treatment, interstitial hydrogen could incorporate into the bond connecting Zn and O and thus changed the electrostatic potential crossing nanorods, which further affected the way electrons moved under UV light illumination and therefore electrons were everywhere instead of staying at the top of nanorods [52]; (2) after hydrogen treatment, oxygen vacancies would increase and thus become the electron capturers to prevent electron–hole recombination, which helped the formation of much more Ag nanoparticles [48]. For ZnO-A@Ag, the formation of many Ag nanoparticles led to the destruction of one-dimensional structure of ZnO-A. This might be due to the formation of oxygen interstitials after air treatment, which became the hole capturers, prevented the electron–hole recombination, and thus enhanced the excess formation of silver nanoparticles. Moreover, considering that the original ZnO crystalline already had oxygen, the crystalline of ZnO nanorods might change after air treatment [53,54]. The EDX analysis revealed that the atomic percentages of silver in the ZnO@Ag, ZnO-H@Ag, and ZnO-A@Ag were 1.28, 3.73, and 8.56, respectively. Obviously, the Ag content of ZnO-A@Ag was the maximum, in agreement with the above observation. In addition, the XRD patterns of ZnO@Ag, ZnO-H@Ag, and ZnO-A@Ag were shown in Figure 2d. As compared to Figure 1d, an additional peak for the (111) plane of silver (fcc) around the scattering angle of 38° was observed for ZnO-A@Ag. This peak was weak or almost invisible for ZnO-H@Ag and ZnO@Ag, respectively, because of the low Ag content. Figure 2e shows the absorption spectra of ZnO@Ag, ZnO-H@Ag, and ZnO-A@Ag. It was obvious that their absorption in the visible light region was increased as compared to Figure 1e. In particular, a broad peak around 430 nm could be observed for ZnO-A@Ag. The above results demonstrated the deposition of Ag nanoparticles on the ZnO nanorod arrays. Considering the uniform deposition and the structural maintenance, ZnO-H was the better support for the deposition of Ag nanoparticles.

**Figure 3:**
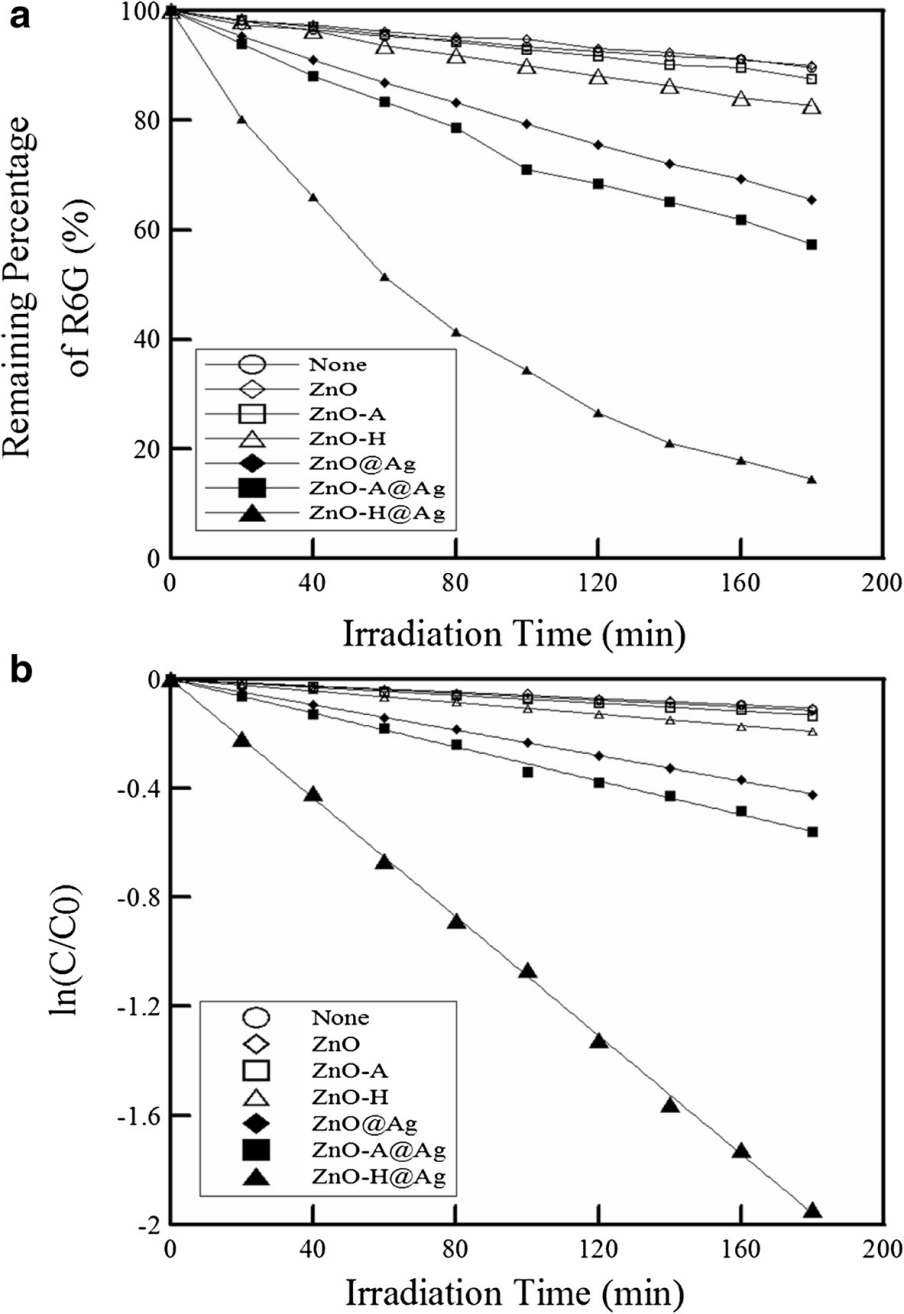
**Photocatalytic degradation of R6G in the visible light region without and with different photocatalysts. (a)** Remaining percentage of R6G vs. irradiation time. **(b)** ln (C/C_0_) vs. irradiation time. Initial R6G concentration at 10^−5^ M; temperature of 25°C.

The photocatalytic degradation of R6G in the visible light region without and with different photocatalysts at an initial R6G concentration of 10^−5^ M and 25°C was shown in Figure 3a. It was obvious that the lowest degradation rate was obtained in the absence of photocatalysts. In the presence of photocatalysts, the degradation rate increased in the sequence of ZnO, ZnO-A, ZnO-H, ZnO@Ag, ZnO-A@Ag, and ZnO-H@Ag. Furthermore, as indicated in Figure 3b, the photocatalytic degradation kinetics was found to follow the pseudo-first-order rate equation [10,55,56], where *C* denotes the concentration of R6G and the subscript 0 means the initial value. The corresponding rate constants (*k*) for the case in the absence of photocatalysts and those in the presence of ZnO, ZnO-A, ZnO-H, ZnO@Ag, ZnO-A@Ag, and ZnO-H@Ag were 5.79 × 10^−4^, 5.82 × 10^−4^, 7.26 × 10^−4^, 1.06 × 10^−3^, 2.33 × 10^−3^, 3.10 × 10^−3^, and 1.09 × 10^−2^ min^−1^, respectively. This revealed that the deposition of Ag nanoparticles on ZnO nanorods efficiently enhanced the photocatalytic activity in the visible light region owing to the extended absorption from UV region to visible light region. Also, ZnO-H@Ag exhibited the maximum photocatalytic ability in the visible light region even if its Ag content was lower than ZnO-A@Ag. The possible reasons were as follows: (1) ZnO-H was the better support for the uniform deposition of Ag nanoparticles and the maintenance of ZnO nanorod arrays, which made the Ag nanoparticles to be utilized efficiently; (2) hydrogen treatment led to the increase of electron mobility, which helped the rapid reaction with molecules and water to form free radicals and enhanced the photocatalytic performance; (3) after hydrogen treatment, the interstitial hydrogen could become shallow donors and therefore the electrons could be excited easily under visible light illumination [57].

**Figure 4:**
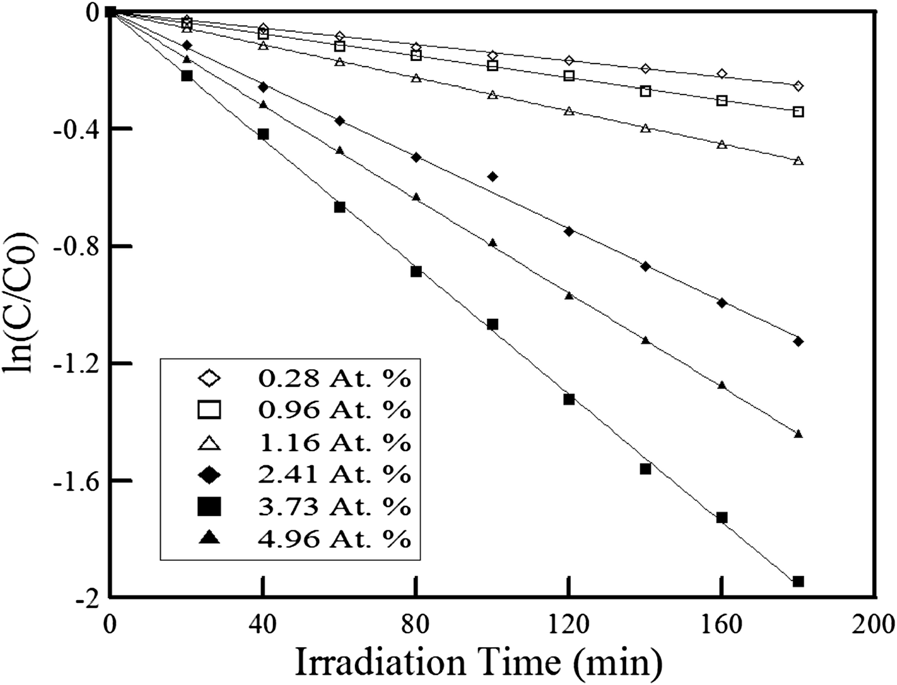
**Photocatalytic degradation of R6G in the visible light region by ZnO-H@Ag with different Ag contents.** Initial R6G concentration at 10^−5^ M; temperature of 25°C.

According to the above discussion, ZnO-H@Ag was used in the following photocatalytic study. First, the effect of Ag content on the photocatalytic activity of ZnO-H@Ag was examined. By conducting the photo-reduction deposition step for 4, 7, 10 min, 10 min × 2, 10 min × 3, or 10 min × 4, the atomic percentages of silver in the resultant ZnO-H@Ag were 0.28, 0.96, 1.16, 2.41, 3.37, and 4.96, respectively. This revealed that increasing the deposition time or repeating time could raise the Ag content. Furthermore, their utilization for the photocatalytic degradation of R6G at an initial R6G con-centration of 10^−5^ M and 25°C was indicated in Figure 4. The corresponding rate constants were obtained as 1.40 × 10^−3^, 1.88 × 10^−3^, 2.81 × 10^−3^, 6.17 × 10^−3^, 1.09 × 10^−2^, and 8.00 × 10^−3^ min^−1^, respectively. It was found that the rate constant increased with increasing the Ag content up to 3.37%. This could be reasonably attributed to the fact that more Ag nanoparticles could absorb more visible light. However, when the Ag content was above 3.37%, the rate constant decreased. Because the catalytic activity depended on the particle size and increasing the repeating time might increase not only the particle number but also the particle size, it was suggested that larger Ag nanoparticles might be formed when the deposition step was repeated for four times and therefore led to the decrease of catalytic activity. In addition, upon illumination, the electrons on silver nanoparticles tended to migrate to the conduction band of ZnO. However, if there were too many silver nanoparticles, the electrons might migrate back to Ag nanoparticles, which formed the recombination centers and lowered the photocatalytic efficiency [58]. Thus, the ZnO-H@Ag with 3.37% of silver was used for the investigation of other factors.

**Figure 5:**
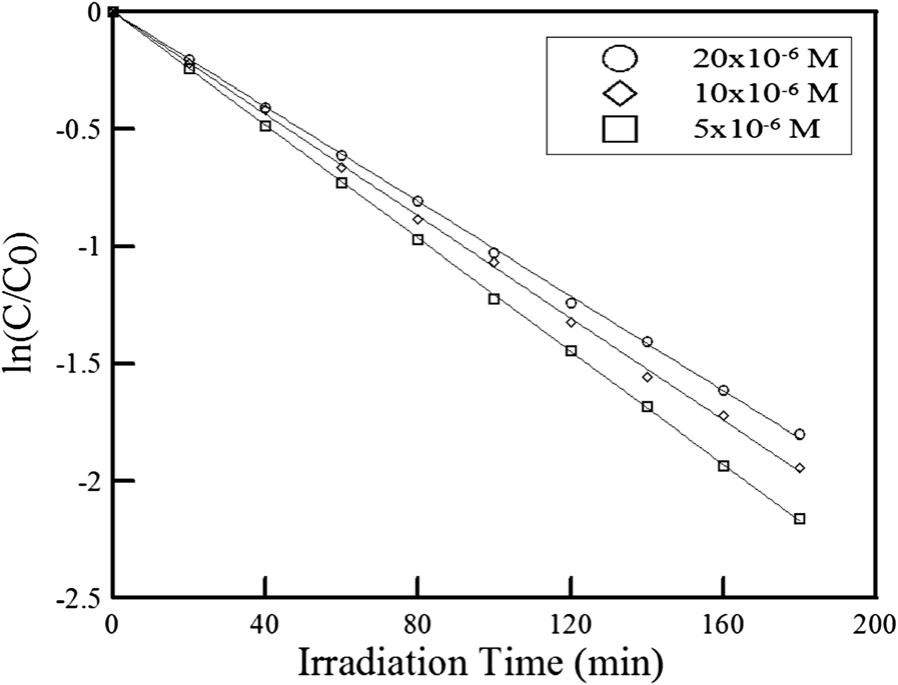
**Effect of initial R6G concentration on photocatalytic degradation of R6G in visible light region by ZnO-H@Ag.** Temperature of 25°C.

The effect of initial R6G concentration on the photocatalytic degradation of R6G at 25°C was shown in Figure 5. The rate constants were 1.20 × 10^−2^, 1.09 × 10^−2^, and 1.01 × 10^−2^ min^−1^ when the initial R6G concentrations were 0.5 × 10^−5^, 1.0 × 10^−5^, and 2.0 × 10^−5^ M, respectively. They have no quite significant differences. Higher initial dye concentration led to only slight decrease of rate constant. This was similar to some previous works [55,59] and could be referred to (1) more dye molecules occupied more active sites on ZnO and (2) the turbidity would increase when the dye concentration became high, which led to the scattering of the incident visible light and therefore lowered the photocatalytic rate.

**Figure 6:**
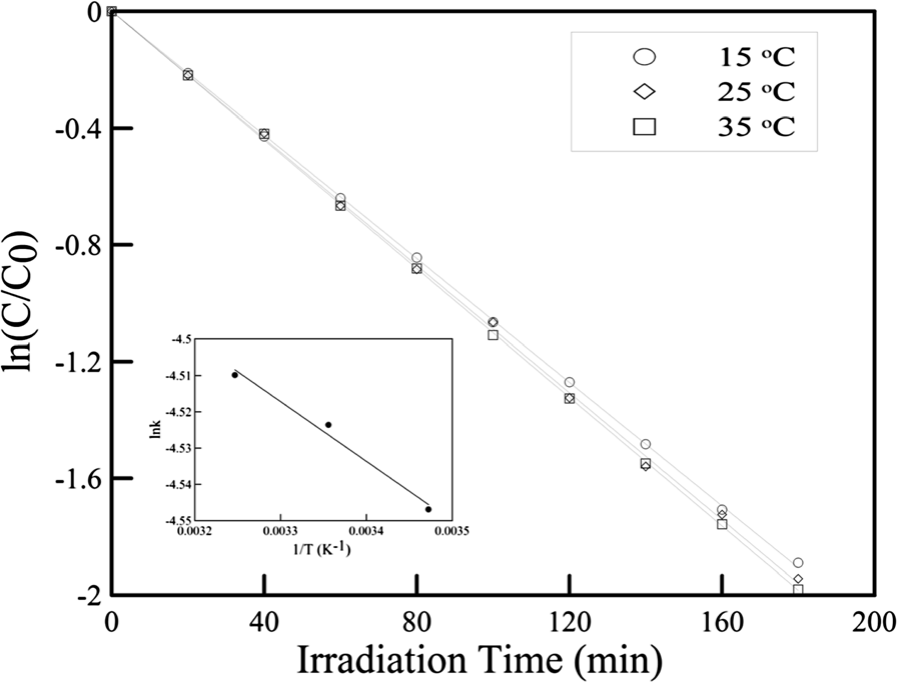
**Effect of temperature on photocatalytic degradation of R6G in the visible light region by ZnO-H@Ag.** Initial R6G concentration at 10^−5^ M. The inset is the corresponding Arrhenius plot.

The effect of temperature on the photocatalytic degradation of R6G at an initial R6G concentration of 10^−5^ M was shown in Figure 6. It was found that the photocatalytic rate increased only slightly with increasing the temperature. This revealed that the increase of temperature slightly helped the photocatalytic reaction to compete with electron–hole recombination more efficiently, leading to an increase in photocatalytic efficiency [53]. The rate constants were 1.06 × 10^−2^, 1.09 × 10^−2^, and 1.10 × 10^−2^ min^−1^ when the temperatures were 15°C, 25°C, and 35°C, respectively. From the Arrhenius plot as shown in the inset of Figure 6, the activation energy was determined to be 1.37 kJ/mol. Such a low value accounted for the weak temperature dependence.

**Figure 7:**
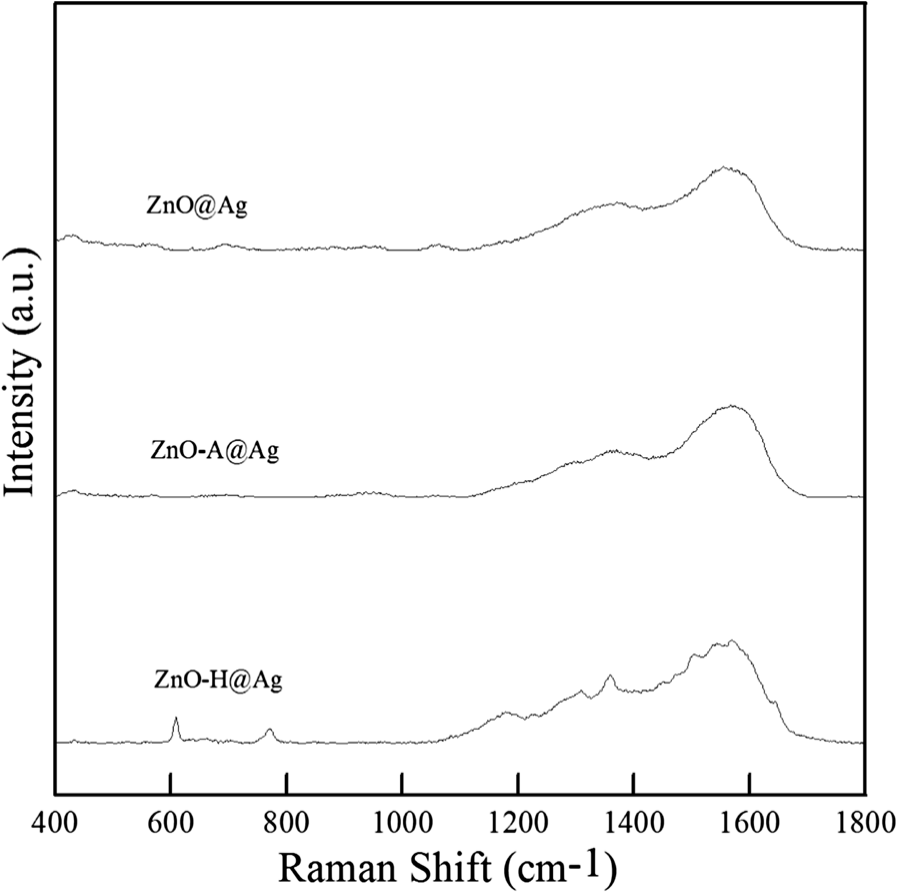
**SERS spectra of R6G on ZnO@Ag, ZnO-A@Ag, and ZnO-H@Ag.** R6G concentration at 10^−9^ M.

R6G was chosen as the target for the study on the SERS property. Its characteristic peaks included 611, 772, 1,178, 1,304, 1,360, 1,503, 1,569, and 1,645 cm^−1^. Figure 7 shows the SERS spectra of 10^−9^ M R6G on ZnO@Ag, ZnO-A@Ag, and ZnO-H@Ag (i.e., the photo-reduction deposition of Ag nanoparticles was repeated for three times). It was obvious that under such a low R6G concentration, the SERS spectrum could be observed clearly only on the ZnO-H@Ag. According to the previous work, Ag nanoparticles exhibited plasmon resonance upon the illumination of visible light, which enhanced the electric fields between nanorods, and thus there formed lots of ‘hot spots’ to enhance the SERS performance [35]. Three kinds of hot spot could be caused: (1) between the Ag nanoparticles on the side surface of the same nanorod, (2) between the two Ag nanoparticles on the side surface of two neighboring nanorods, and (3) between the two Ag nanoparticles on the tops of two neighboring nanorods [35]. In this work, Ag nanoparticles were uniformly deposited on the top, side, and bottom of the ZnO nanorods for ZnO-H@Ag, which possessed all the above kinds of hot spots and exhibited the best SERS property. ZnO@Ag had small and little Ag deposition only on its top, which barely formed any kind of hot spot and therefore its SERS property was poor. For ZnO-A@Ag, the deposition of lots of Ag nanoparticles led to the structural destruction of ZnO nanorod arrays, which could not form effective electric fields and therefore, its SERS property was also poor.

**Figure 8:**
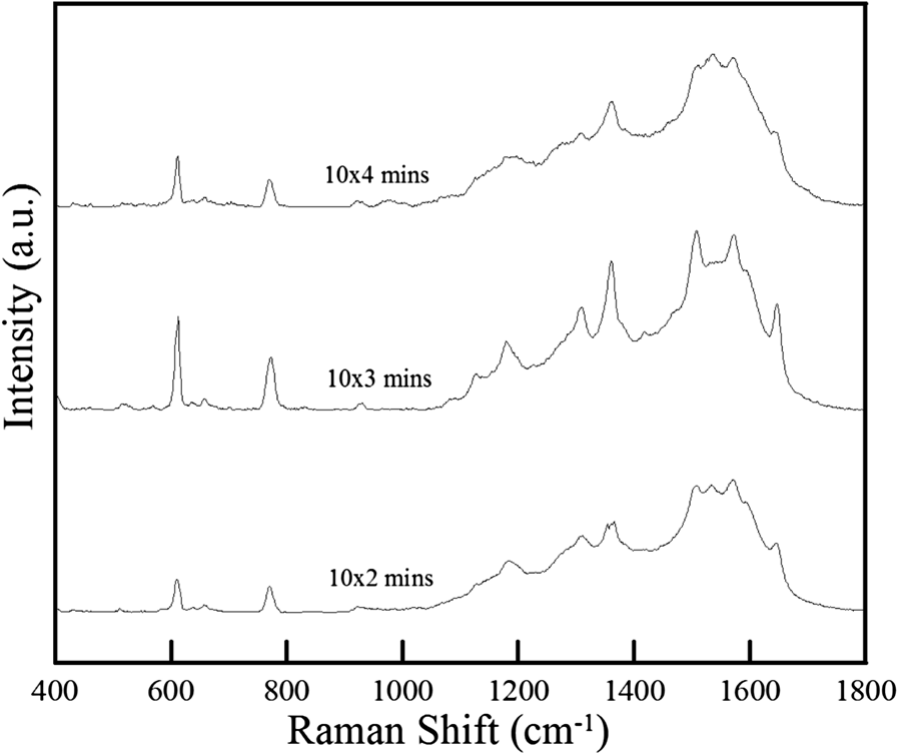
**SERS spectra of R6G on ZnO-H@Ag obtained by repeating the Ag nanoparticles deposition for different times.** R6G concentration at 10^−9^ M.

**Figure 9:**
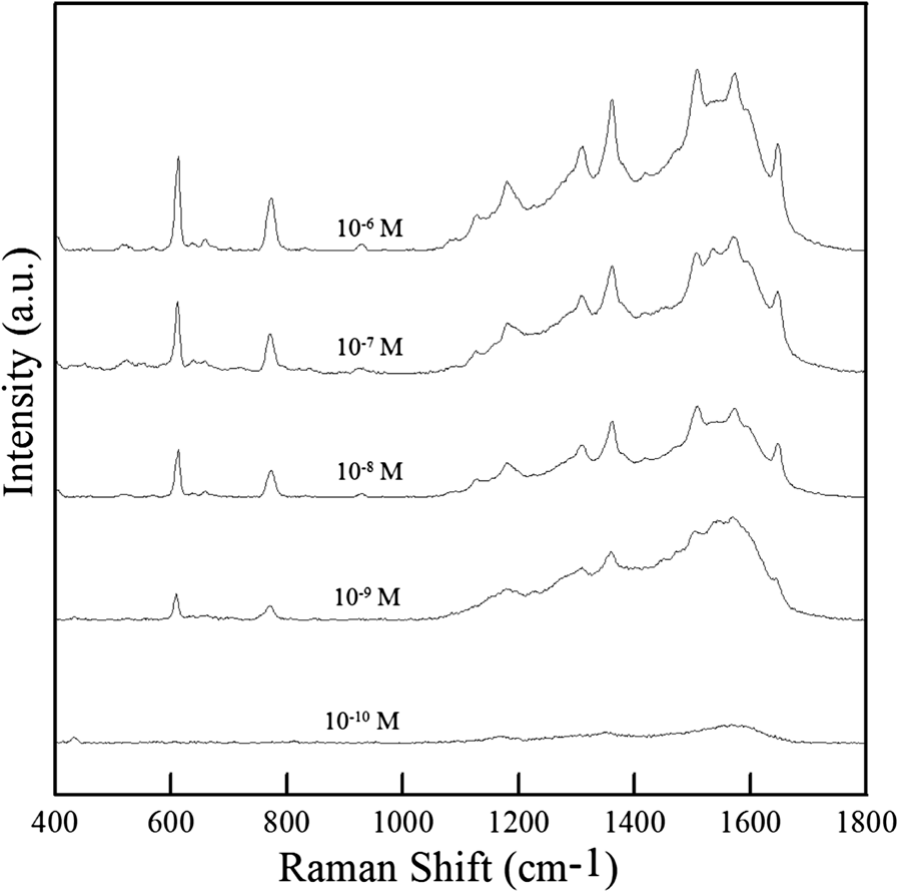
SERS spectra of R6G on ZnO-H@Ag at different R6G concentrations.

Moreover, using the ZnO-H@Ag obtained by changing the repeating time to 2 or 4, the intensity of SERS spectra was decreased as indicated in Figure 8. This revealed that the ZnO-H@Ag obtained at a repeating time of 3 was the better substrate for the SERS application. When the repeating time was 2, fewer hot spots would be formed because of the presence of less Ag nanoparticles. When the repeating time was 4, the slight agglomeration of Ag nanoparticles occurred (particularly on the tops of nanorods) and led to the decrease of SERS intensity. Accordingly, the ZnO-H@Ag obtained at a repeating time of 3 was further used for the SERS analysis of R6G at different concentrations (10^−6^ ~ 10^−10^ M). As shown in Figure 9, when R6G decreased from 10^−6^ to 10^−9^ M, the main characteristic peaks at 611, 772, and 1,360 cm^−1^ still could be observed. However, when R6G concentration decreased to 10^−10^ M, the characteristic peaks became invisible. Therefore, the detection limit of R6G for ZnO-H@Ag was 10^−9^M.

## Conclusions

In this work we have successfully synthesized Ag-coated ZnO nanorod arrays for the photocatalytic degradation and SERS analysis of R6G. Hydrogen treatment of ZnO nanorod arrays was demonstrated to be useful for the uniform deposition of Ag nanoparticles on the top, side, and bottom of ZnO nanorods. As compared to ZnO@Ag and ZnO-A@Ag, the ZnO-H@Ag showed the better photocatalytic activity for the degradation of R6G in the visible light region. Also, the photocatalytic degradation of R6G obeyed the pseudo-first-order kinetics, and the optimal atomic percentage of silver in ZnO-H@Ag was 3.37. With decreasing the initial R6G concentration or increasing the temperature, the corresponding rate constant increased slightly. The activation energy was 1.37 kJ/mol. In addition, the ZnO-H@Ag with an Ag atomic percentage of 3.37 was also demonstrated to be the best one for the SERS analysis of R6G as compared to ZnO@Ag, ZnO-A@Ag, and the ZnO-H@Ag with other Ag contents. The detection limit of R6G was 10^−9^M. The whole result revealed that hydrogen treatment of ZnO nanorod arrays was useful in improving the uniform deposition of Ag nanoparticles on ZnO nanorod arrays, which led to better visible-light photocatalytic and SERS properties.

## Competing interests

The authors declare that they have no competing interests.

## Authors’ contributions

SLL carried out the experiments and drafted the manuscript. KCH carried out the measurement of SERS spectra. CHH provided the assistance in the preparation of ZnO nanorod arrays. DHC guided the study and modified the manuscript. All authors read and approved the final manuscript.

## Authors’ information

SLL received his master degree in Chemical Engineering at National Cheng Kung University (Taiwan) in 2012 and now is in the army. KCH is currently a PhD student of the National Cheng Kung University (Taiwan). CHH received his PhD degree in Chemical Engineering at National Cheng Kung University (Taiwan) in 2011 and now works as a researcher in United Microelectronics Corporation (Taiwan). DHC is a distinguished professor of Chemical Engineering Department at National Cheng Kung University (Taiwan).
